# Porous Cellulose Substrate Study to Improve the Performance of Diffusion-Based Ionic Strength Sensors

**DOI:** 10.3390/membranes12111074

**Published:** 2022-10-29

**Authors:** Hamid Khosravi, Pouya Mehrdel, Joan Antoni López Martínez, Jasmina Casals-Terré

**Affiliations:** 1 Mechanical Engineering Department—MicroTech Lab., Universitat Politècnica de Catalunya (UPC), C/Colom 7-11, 08222 Terrassa, Barcelona, Spain; 2 Department of Mining, Industrial and ICT Engineering (EMIT), Universitat Politècnica de Catalunya (UPC), AV. Bases de Manresa 61-73, 08240 Manresa, Barcelona, Spain

**Keywords:** microfluidic paper-based analytical devices, quantitative assay, flow behavior, cellulose-based membrane, diffusion-based sensor, sisal paper

## Abstract

Microfluidic paper-based analytical devices (µPADs) are leading the field of low-cost, quantitative in-situ assays. However, understanding the flow behavior in cellulose-based membranes to achieve an accurate and rapid response has remained a challenge. Previous studies focused on commercial filter papers, and one of their problems was the time required to perform the test. This work studies the effect of different cellulose substrates on diffusion-based sensor performance. A diffusion-based sensor was laser cut on different cellulose fibers (Whatman and lab-made Sisal papers) with different structure characteristics, such as basis weight, density, pore size, fiber diameter, and length. Better sensitivity and faster response are found in papers with bigger pore sizes and lower basis weights. The designed sensor has been successfully used to quantify the ionic concentration of commercial wines with a 13.6 mM limit of detection in 30 s. The developed µPAD can be used in quantitative assays for agri-food applications without the need for any external equipment or trained personnel.

## 1. Introduction

The advantages of miniaturizing fluid handling are appreciated in different sectors, such as in food, agri-food industry, or in biochemical, biomedical, and pharmaceutical applications [1,2,3,4]. This novel technology, thanks to its low reagent requirement and fast responsiveness, encourages both scientists and entrepreneurs to develop new devices and progress its applicability to alternative fields, particularly in the Micro Total Analysis Systems (µTAS) [5,6,7,8,9]. There are still barriers to overcome such as accuracy compared to conventional processes or their user-friendliness. Typically, extremely efficient microfluidic platforms depend on external energy sources to manage the flow or the incorporation of sophisticated detection methods. Therefore, the in-situ determinations or the utilization in less developed countries is seriously restricted. Whitesides in 2006 [10] proposed to merge the advantages of microfluidics with the benefits of the capillary flows in porous substrates. This idea has been implemented in different research labs that have started the development of microfluidic paper-based analytical devices (µPAD) [11,12,13,14]. However, the fact that the capillary flow relies on the paper features, complicates the flow control. Currently, most µPADs are used for qualitative monitoring, otherwise, they still need sophisticated detection approaches [15,16].

Porous media are matrices of different types of fibers, mainly, cellulosic fibers. In particular, filter papers, due to their well-known maximum pore size, are the ones most commonly used by researchers. The goal is to achieve repeatability in the wicking capacity and stability in flow generation. Thus, the most common approach is to use Whatman papers when designing processes [17] or devices [18]. The current research efforts are either in modifying the paper surface or studying the paper manufacturing process. In this regard, remarkable µPADs have been developed that prepare quantitative or semi-quantitative detections without the need for complex procedures, such as K^+^ or troponin [19,20]. On the other hand, different manufacturing methods have been developed to produce µPADs from Whatman filter paper [21,22,23], methods that can be easily implemented in developing regions. Researches done on paper-based microfluidics are currently enhancing the resolution of the sensors [24,25,26].

There are authors starting to modify the flow rate using stacks of different papers [27]. Other authors have explored different types of fiber matrices to completely change the fluidic behavior. For instance, the use of paper towels as wicking pads [28], which is cheaper and provides a higher flow rate but is less accurate. Other approaches use nitrocellulose membranes, which offer improved binding capabilities due to the available functional groups, providing the possibility to implement low-cost ELISA-like tests [29]. Some works have taken the benefits of fiber-glass platforms to immobilize nanoparticles and improve their surface-to-volume ratio [30,31,32]. This can successfully increase their repeatability and reliability of detection. Nitrocellulose and fiber-glass membranes provide more accuracy and repeatability, but they are not as environmentally friendly as cellulose matrices.

Therefore, there is a need to enhance and understand the flow behavior of cellulose-based membranes in order to achieve accurate quantitative measurements based µPADs (by non-trained personnel). Currently, commercial success relies on easy-to-detect molecules such as paper-based pregnancy tests, in which the marker agent is immobilized within the porous media [11] and the results do not need to be quantitative. Some works have utilized paper-based sensors to quantify different compounds through different analytical techniques, for instance, electrochemiluminescence (ECL) [33], chemiluminescence (CL) [34], and electrochemical (EC) [35]. These determination approaches enhance the accuracy of tests and extend the horizon of applicability, but they require flow stability to provide accuracy.

The employment of the above-mentioned techniques does not help in the simplification of the analysis to apply µPADs, since sometimes power sources or potentiostats are required. In the pioneering studies, the effect of the substrate itself (paper parameters) on the efficiency of devices was not investigated. Now, researchers have realized that efforts need to be made on understanding the effects of paper itself on the sensor’s performance. Hertaeg et al. [36] have considered the effect of papers on the sample pre-treatment step on the diagnosis (basically on red blood cell trapping), but not in the analysis step. Casals et al. [37,38] also pointed out that in the pre-treatment step, the use of environmentally friendly fibers such as sisal provided promising results if the characteristics of the paper were fully understood.

In the measurement step, a sequential flow of the analytes to the pre-treated detection zone is required. To achieve the appropriate sequential arrival of the analytes, careful control of the flow rate is needed in the porous matrix. The approaches to managing the flow can be classified into three categories: mechanical-based, chemical-based, and geometrical-based [39,40]. For three-dimensional µPADs, the mechanical-based flow control is achieved by a set of mechanical valves, which connect between paper substrates at various levels and guide the flow [21]. The chemical-based flow control depends on the adjustment of the porous substrate features when the analytes reach the pores (voids) [41]. Alternatively, there are geometrical methods based on the alteration of the channel width, length, or any obstruction to manage the fluid arrival to the detection region [42]. Another geometrically-based approach relies on the creation of a paper network by consecutively placing the fluid source closer to the channel. The closest source moves the fluid faster than the other sources, enabling the delivery of reagents to the detection zone in a programmed manner [43]. Alternatively, Toley et al. [44] suggested diverting the fluid into an absorbent pad, a shunt, to delay the fluid flow. However, the previous approaches are suitable for sequential flow analyses, and they do not enable flow control in applications in which co-laminar flows are demanded.

Su et al. [45] and Li et al. [46] moved from commercial filter paper conventionally used in paper-based microfluidics to the study of lab-made papers and their characteristics. Both research groups pointed out that for blood typing analysis the pore size and wood fiber type are two important parameters. However, blood typing tests are a type of test where the results are qualitative or in other words, you determine a given number of blood types but not in a quantitative manner. Casals et al. [37] went one step further and focused on nonwood-based lab-made papers, first to see which other parameters of the paper manufacturing process also influenced the qualitative test (or optical detection process), and secondly to evaluate if nonwood-based papers were equally valid to perform such test with the advantage of short harvesting times and therefore more sustainable manufacturing process. In their papers, the analytical performance of sensors manufactured with lab-made papers with different basis weights and refining were evaluated. The other characteristics of the resulting papers were also evaluated (density, thickness, and capillary rise) and their influence on the performance was analyzed.

Later, Mehrdel et al. [47] proposed to use a diffusion-based sensor made out of paper as a substrate. This new strategy allows the quantification of the analyte and not only the detection of its presence. They focused on the study of co-laminar flows on paper-based microfluidic platforms and presented for the first time a computational fluid dynamics model capable of taking into account the influence of paper properties (fiber length, fiber diameter, density) and geometry of the substrate on the flow characteristics, particularly on the diffusion. The model validation was done using only a single type of fiber (cellulose) and commercial filter papers (Whatman 5). One of the limitations of the proposed device was the turnaround time, which is excessive for point-of-care applications.

In this paper, lab-made papers from nonwood cellulose fibers, with different paper characteristics without any added chemical additive were used to analyze the effect of different parameters on the response time and the performance enhancement in diffusion-based sensors. The paper is organized as follows: the methods and materials describe the sensor principle of operation, a computational fluid dynamic model used to forecast the most suitable paper substrates. Finally, the paper substrates are manufactured and used to validate the results. The results section describes the results achieved using the ionic concentration detection system to analyze wine samples and presents the characteristics of the porous substrate that minimize the use of pre-treated substrate/particles and alternatively enhance the diffusion of media and accordingly accelerate the detection time.

## 2. Materials and Methods

### 2.1. Sensor Principal of Operation

**Figure 1:**
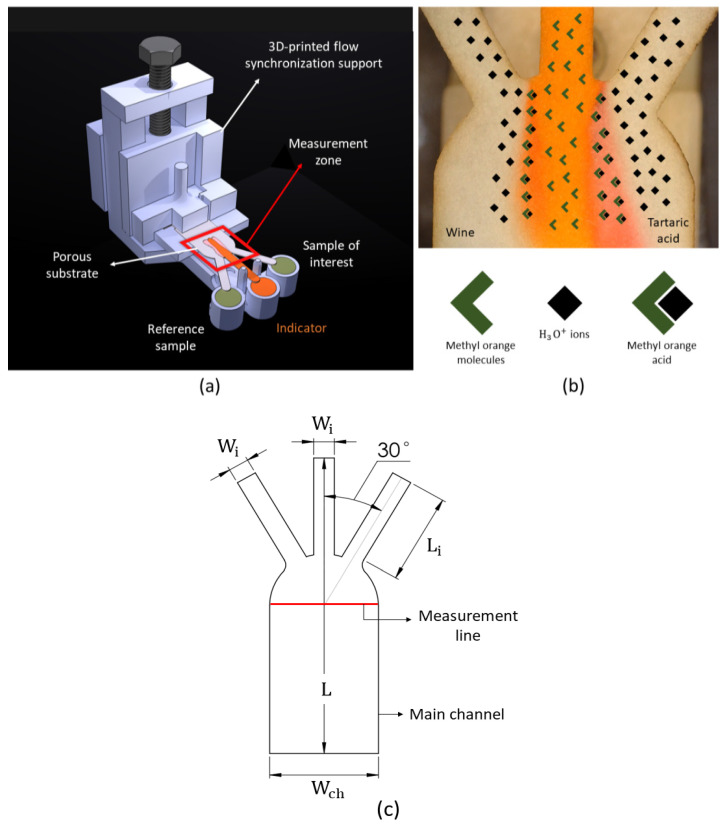
(**a**) Schematic of the μPAD ionic concentration sensor parts. (**b**) Physics of the ionic concentration measurement. (**c**) Porous substrate geometry.

In this paper, we use a paper diffusion-based microfluidic sensor. Mehrdel et al. [47] proposed the use of this sensor for ion concentration and developed a 3D-printed support to achieve the synchronization of the fluid in the three different inlets. This type of sensor requires three channels: one for the sample of interest, one for the indicator (here as a pH indicator), and third for the reference sample (tartaric acid). The hydronium ions $\left[H_{3}O^{+}\right]$ from the sample of interest and the reference, diffuse into the indicator, which changes its color. The color shift is due to the transformation of the methyl orange molecules from Azo structure to Quiniod structure. The number of indicator molecules that change color depends on the number of ions that diffuse into it. Therefore, the width of the color can be correlated to the ion concentration, see Figure 1b. The main challenge is flow synchronization, especially in capillary substrates such as papers. Hence, Mehrdel et al. [47] introduced a 3D-printed support that ensured simultaneous contact of all the reagents with the porous membrane; see Figure 1a for how this problem was solved.

According to results from Mehrdel et al. [47], the flow stabilizes faster if the angle between the inlets is 30 degrees, therefore, the performance of the diffusion-based sensor will be enhanced. Table 1 summarizes the substrate dimensions selected in this investigation. The concentration results are obtained by comparing the diffusion widths at the measurement line between the sample of interest and the reference, which is shown as the measurement line in Figure 1c.

### 2.2. Sensor Materials

#### 2.2.1. Porous Membrane Substrate

Different Whatman filter papers (from @Fisher Scientific, Hampton, NH, USA) and lab-made Sisal papers (with different basis weights) are used as the assay substrate.

The lab-made Sisal paper sheets were produced from elemental chlorine-free bleached sisal fibers. Sisal pulp (S-pulp) was obtained from CELESA mill in (Tortosa Spain). Initially, the pulp was disintegrated at 30,000 revolutions (S0 pulps), and then, in a PFI mill, one aliquot of pulp was refined at 1000 rpm (S1000) following procedures from standards ISO 5263 and ISO 5264, respectively. Refining, modified the fiber surface and increased the inter-fiber bonding capacity, therefore, the paper network changed, achieving better sheet formation and enhanced mechanical properties. Fiber length and percentage of fines were measured according to TAPPI standard method T271 using a Kajaani FS300 fiber analyzer. Rapid-Köthen method was used to prepare lab sheets, according to ISO 5269, at different basis weights from each unrefined and refined fiber sample, S0_50 being 50 g/m^2^ and S0_100 being 100 g/m^2^.

Table 2 summarizes the geometrical and physical characteristics of cellulose fibers, present in Whatman and lab-made Sisal papers.

#### 2.2.2. Reagents

Tartaric acid (2,3-Dihydroxybutanedioic acid) was purchased from @Merck Schuchardt OHG (Hohenbrunn, Germany). Three concentrations of 0.1, 0.5 and 1.0 M of tartaric acid were prepared. The pH values of the prepared tartaric acid solutions at RT were 2.05, 1.56 and 1.29, respectively. Methyl orange (Sodium 4-{[4-(dimethylamino)phenyl]diazinyl}benzene-1-sulfonate) which is used as pH indicator, was prepared by dissolving 40 mg of it in 40 mL of water. Due to its pH transition rate (turns to red at pH < 3.0 and yellow at pH > 4.0), it is suitable for the expected pH value of wines and tartaric acid solutions (at or lower than pH 3.7) [48]. Commercial white wine was purchased for validation of the sensor performance.

Table 3 summarizes the principal physical properties of the reagents used in this experiment.

### 2.3. Numerical Simulation

#### 2.3.1. Fluid Flow and Diffusion Phenomena

Mehrdel et al. [47] used computational fluid dynamics (CFD), Ansys fluent software to model the flow in a porous substrate such as the one plotted in Figure 1c. The model is valid for incompressible, Newtonian fluid and isothermal processes. The model, due to dimensions works on the laminar regime, used a couple of schemes to solve the following Navier–Stokes equation:

(1) $$\nabla \vec{U}=0,$$

(2) $$\rho \vec{U}\;.\;\nabla \vec{U}=-\nabla P+\mu \nabla^{2}C$$

where $\vec{U}$, ρ, ∇P and, C are the velocity vector, density of the working fluid, the pressure gradient, and the species concentration within the solving domain, respectively.

The equation for convection–diffusion is defined as:

(3) $$\rho \vec{U}\;.\;\nabla C=D\nabla^{2}C$$

where D is the diffusion coefficient of the species.

Equation (3) couples the convection–diffusion transport phenomena under the laminar regime and it is introduced in the model as user-defined scalar. In this study, the specie that diffuses is the proton $H^{+}$ with a diffusion coefficient of $D=7\times 10^{-9}$ m^2^/s [50]. Since the diffusion occurs on a porous media, the model uses an effective diffusion coefficient $\left(D_{eff}\right)$ [51]:

(4) $$D_{eff}=D_{0}\;\varepsilon$$

where ε is the porosity and $D_{0}$ is the diffusion coefficient in [kg/m.s], obtained by multiplying the diffusion coefficient of $H^{+}$ molecule (D) and the density of the fluid (ρ).

The porous substrate is modeled through the following parameters: the porosity (ε) (in other words, void fraction) and the permeability (α) of the porous substrate. The porosity can be calculated as $\varepsilon =1-\rho_{p}/\rho_{c}$ where $\rho_{p}$ is the density of the whole porous substrate and $\rho_{c}$ the density of its cellulose fibers. The permeability (α) relates to the physical and geometrical properties of the substrate, and it is calculated through the following equation:

(5) $$\frac{\alpha }{d^{2}}=\frac{\varepsilon^{3}}{\varphi {\left(\frac{L}{L_{f}}\right)}^{2}{\left(1-\varepsilon \right)}^{2}}$$

where $L_{f}$, L, d and, φ are the fiber’s length, substrate’s total length, fiber’s diameter, and the pore shape factor, respectively.

The pore shape factor for 0.4 < ε < 0.9 is equal to φ=140 [52]. Table 4 summarizes the parameters of the papers that were used in the model.

#### 2.3.2. Model Boundary Conditions

The working fluid is assumed to be liquid water under standard room conditions (RT = 25 °C and HR = 50%) with a density and viscosity of 998.2 kg/m^3^ and 0.001003 kg/m.s, respectively. At the inlets of the lateral channels, the initial species concentration is set to 0.05 M.

The velocity is defined at the inlets. The initial flow velocity at the inlets is estimated from previous capillary flow characterization done with the different paper substrates, measuring the time required to fill the inlet branches, see Table 5. The fact that the void spaces decrease after wetting (fiber’s swelling) is not considered in this model.

The convergence residuals are set to 1 $\times \;10^{-8}$ for all the criteria. The viscous resistance is obtained for each different porous substrate from the inverse of the permeability [47]. The diffusion constant, velocity and viscous resistance (1/permeability) for each different paper are summarized in Table 5.

**Figure 2:**
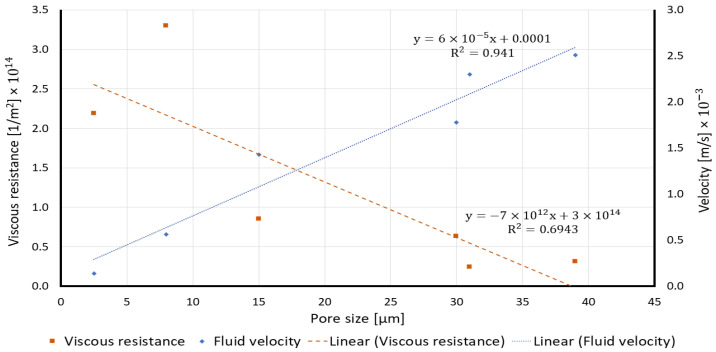
Effect of the pore size on the viscous resistance and fluid velocity in porous media.

Based on the measured fluid velocities and calculated viscous resistance in all the paper substrates, Figure 2 plots the effect of the average pore size on the viscous resistance and velocity through a porous medium.

Figure 2 illustrates that there is a reverse relation between the fluid velocity and viscous resistance through the porous medium of a paper. As the average pore size increases, the fluid velocity through the porous medium increases which causes a reduction in the viscous resistance.

Owning to the fact that the investigated geometry is identical to the conducted study by Mehrdel et al. [47], therefore, “Fine” mesh distribution with an element size of 50 µm and a minimum surface area of $1.93\times 10^{-5}$ (m^2^) is used for the numerical analysis.

### 2.4. Experimental Setup

**Figure 3:**
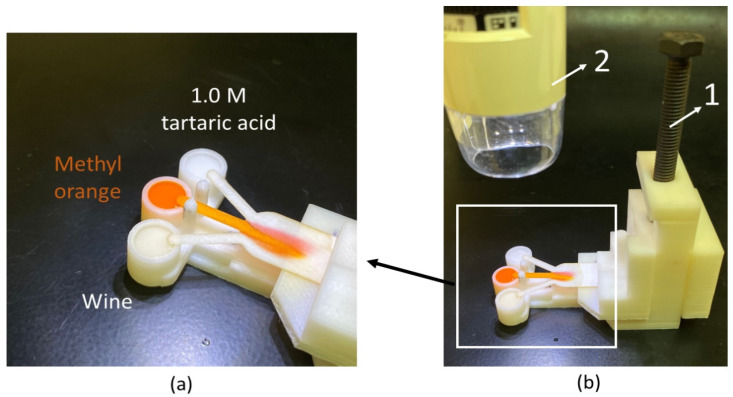
(**a**) Picture of the ionic strength wine evaluation. Each reservoir is filled with 60 µL of solution. (**b**) Picture of a test under performance. Numbers 1 and 2 are the vertically adjustable arm and digital microscope, respectively.

The 30-degree model with a 3-inlet substrate was laser cut (using a NEJE7000mW laser) in the different paper substrates. The 3D-printed support described in [47] was used to allow the three inlets of the paper strip to encounter the reagents simultaneously, thus minimizing the human error factor (synchronization errors) and guaranteeing repeatability. All the reservoirs are filled with 60 µL of solution (Figure 3a) and by taking advantage of the vertically adjustable arm, the inlets contacted the reservoirs at the same time (number 1 in Figure 3b).

Since the diffusion width varies along the length of the sensor (see Equation (3)), it is necessary to establish a benchmark time in order to obtain repeatable and accurate results.

A Dino-Lite MS325B microscope is used to take pictures of the defined measurement line (number 2 in Figure 3b).

ImageJ processing software is used to evaluate the diffusion width over the measurement line. The diffusion width is measured by RGB profile analysis tool. This tool evaluates the intensity change in the green scale when the reaction between the reagents and the pH identifier occurs. The moment that the intensity of the green channel starts to drop (the beginning of the reaction) is set as “Time zero”. The pictures for analysis are taken at 30 s, 60 s and 120 s after the “Time zero”. The ambient and the projected light are the same in all the assays.

#### Errors and Data Curing

The errors in this work might have occurred during numerical simulations and experimental sections. To ensure that the mathematical method is as close as possible to the real flow, careful efforts have been made, such as considering the effect of discretization and choosing the cells’ size accurately to not affect the final results, as well as choosing the residuals of the results between consecutive iterations correctly to avoid producing a false convergence.

In the experimental section, the errors might have been rooted in mistakes done by staff or devices. To minimize these errors, every assay was repeated at least 5 times for every configuration of solutions on each paper substrate.

Furthermore, careful efforts were made to make sure that all the assays were carried out at similar temperatures, ambient light, and magnification rates.

Finally, by considering the generated standard deviation from the measured diffusion width and regression line, which can be calculated as, y=mx+b, where m is the slope of the regression line and b is the interception point of the line with the *Y*-axis, the limit of detection (LOD) can be found through the following formula:

(6) LOD=3.3×Sm

where S is the standard deviation of the measured diffusion width and m is the slope of the regression line between the diffusion width and concentrations.

## 3. Results and Discussion

Based on the characteristics of the cellulose fibers, a computational fluid dynamic model is evaluated for each paper substrate, and the results obtained from the model are then validated experimentally.

### 3.1. Numerical Results

**Figure 4:**
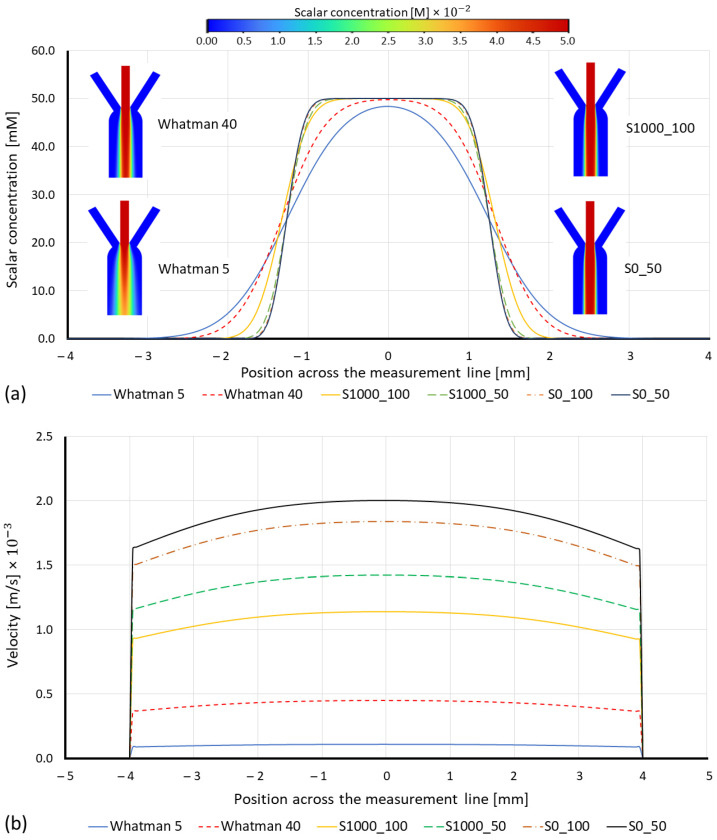
(**a**) Numerical simulation results of the species diffusion width with respect to the papers’ substrate, and (**b**) fluid velocity across the measurement line for different paper substrates.

The flow velocity and the diffusion width in the porous medium for all the paper substrates based on their cellulose fibers’ characteristics are numerically obtained from the steady-state results of the simulations. There are various parameters that influence the motion of fluid in porous media. For instance, Whatman papers, because of the higher density and lower length of their cellulose fibers, showed more fluidic resistance in their matrix compared to Sisal papers. Therefore, the species in Whatman’s paper can diffuse more, see Figure 4a. The diffusion width is wider in Whatman papers compared to Sisal papers, however, due to fluidic resistance, more time and space are needed to achieve this diffusion.

On the other hand, Figure 4b shows how higher viscous resistance causes a considerable reduction in fluid velocity, therefore, at steady-state, the fluid molecules would have more time to diffuse (Whatman papers), but at the same time, in Whatman papers, the flow takes a long time to reach to the measurement time and start the diffusion. Therefore, papers with lower fluidic resistance (lab-made Sisal papers) can start the diffusion process before and obtain measurable data in less time. 

Numerical results obtained by steady-state simulations revealed that Whatman papers showed a wider diffusion width than Sisal papers, while the fluid velocity over the X-direction (measurement line) in Whatman papers was less than in the Sisals. Therefore, to have a comparison between these papers in terms of the time needed to have a measurable diffusion width at the measurement line, the time transient simulation is studied.

**Figure 5:**
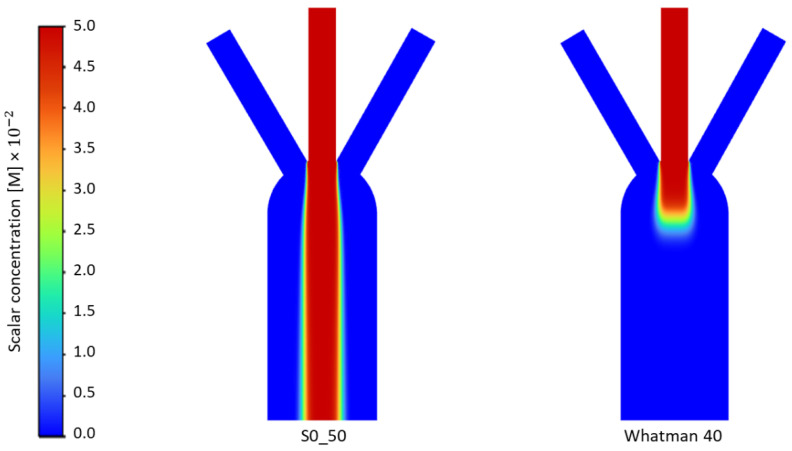
Diffusion contours of Sisal (0_50) and Whatman 40 for time transient simulation.

Figure 5 plots the diffusion contours obtained from time transient results of the simulations for both Whatman and Sisal papers under the same conditions.

### 3.2. Experimental Results

#### 3.2.1. Time to Achieve One Millimeter Diffusion Width

**Figure 6:**
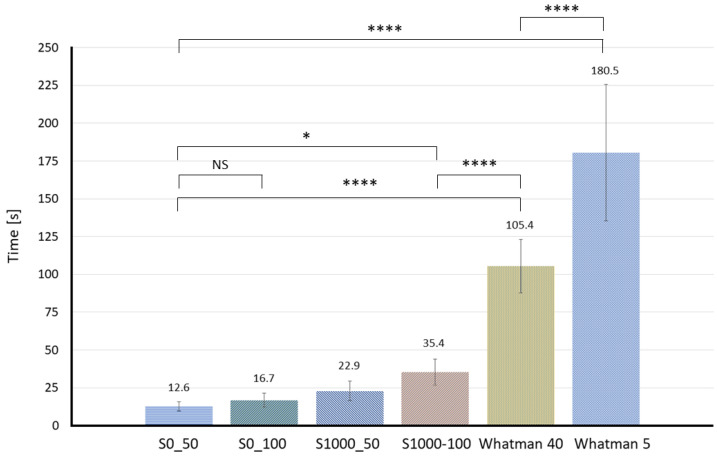
Required time for each different paper substrate to achieve a 1.0 mm diffusion width at the measurement line. The asterisk (*) represents the *p*-value of the statistical test. (* *p* < 0.05, **** *p* < 0.0001, NS: non-significance).

Several paper substrates are prepared and tested (at least 5 tests for every concentration of solutions on each paper substrate). One millimeter diffusion width can be easily measured in the portable experiment setup shown in Figure 3. Therefore, the time required to develop such diffusion width (1.0 mm) was monitored and compared between the different types of substrates. Figure 6 plots the time results for each paper substrate.

**Figure 7:**
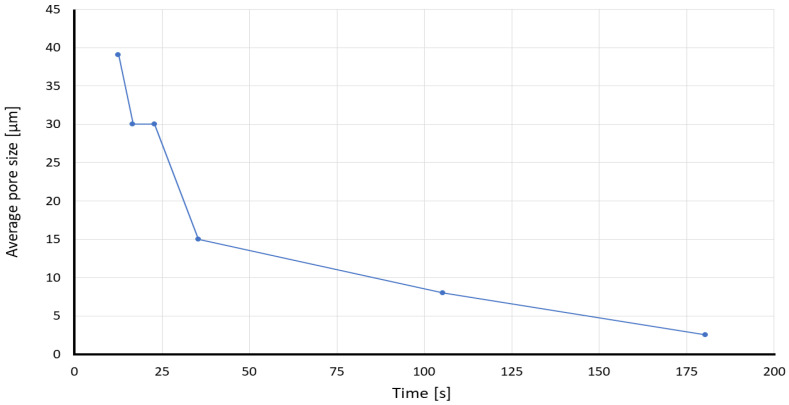
Plot of the average pore size and the time required to develop 1.0 mm diffusion width.

According to the results plotted in Figure 6, lab-made Sisal papers develop a 1.0 mm diffusion width in 85% less time than Whatman papers in general. This can be mostly due to the pore size since as shown in Figure 7, as the average pore size decreases, much time is needed to see the diffusion happens. The commercial Whatman papers have a smaller average pore size and lower permeability compared to lab-made Sisal papers. For instance, the required time for developing 1.0 mm of diffusion width increases from (12.6 ± 3.04 s) in the S0_50 to (180.5 ± 45.85 s) in the Whatman 5. In case of similar average pore sizes (i.e., Sisal 0_100 and 1000_50), the paper strip with less density showed a faster response to develop a measurable diffusion width. (Video S1 shows a comparison of the diffusion development in S0_50 and Whatman 40 papers).

#### 3.2.2. Influence of Pore Size and Viscous Resistance on the Sensor’s Sensitivity

**Figure 8:**
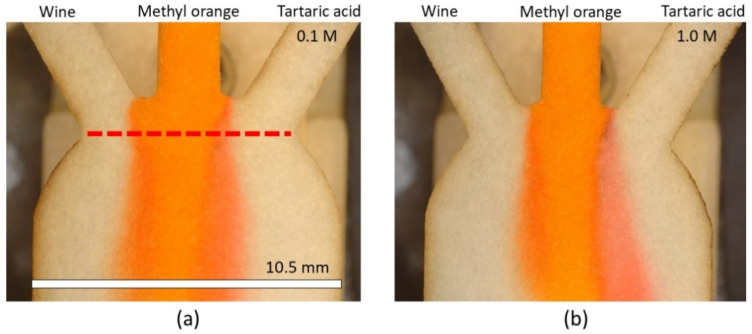
Visual comparison of diffusion development in Whatman 40 paper substrate at 120 s after the “Time zero” for (**a**) 0.1 M and (**b**) 1.0 M of tartaric acid. The measurement line is shown as the dashed line with 7.5 mm of width.

Due to the small pore size and low permeability of Whatman grade 5, Mehrdel et al. [47] studied the diffusion width and limit of detection over a measurement line placed at the inlets’ intersection (as shown in Figure 8a). Moreover, the time to characterize the response of the sensor was selected at 120 s after the “Time zero”. If the response is measured before 120 s, the diffusion width is not clear, and the response of the sensor loses accuracy.

Hence, to have a comparison between two Whatman filter papers (5 and 40), the interaction between the pH indicator and the reagents is analyzed at 120 s after the “Time zero” for Whatman 40, as well. Figure 8 plots a visual comparison of the diffusion development in Whatman 40 for two different tartaric acid concentrations (0.1 M and 1.0 M). In all the pictures, methyl orange enters through the middle inlet, wine through the left inlet, and tartaric acid via the right inlet.

**Figure 9:**
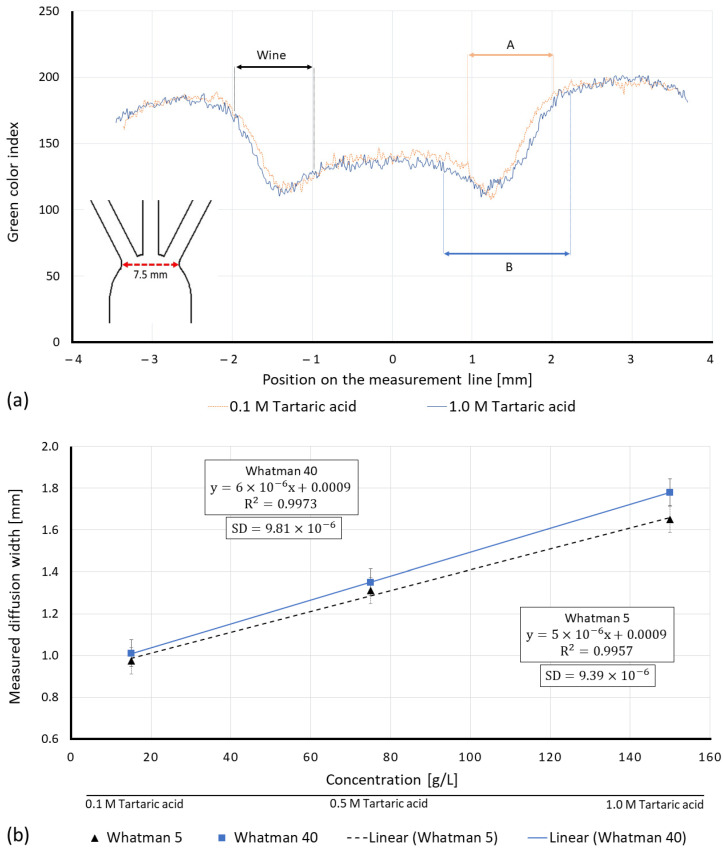
(**a**) Intensity of the green color channel at the measurement line of different tartaric acid concentrations at 120 s after the “Time zero” for the Whatman grade 40 paper strip. (**b**) Comparison between the measured diffusion width based on the changes in the intensity of the green channel and the calibration plots of the Whatman 40 (this work) and the Whatman 5 (previous work) [47] paper substrates. (SD = standard deviation).

Figure 9a shows the intensity of the green color of Whatman grade 40 at the measurement line (red dashed line) for both 0.1 M and 1.0 M of tartaric acid as references. The measured diffusion width of wine remained constant and showed a diffusion width of 980 ± 12.6 µm, while, for the 0.1 M and 1.0 M of tartaric acid, it increased from 1012 ± 57.4 µm (arrow A) to 1781 ± 65.2 µm (arrow B), respectively, since 1.0 M tartaric acid has higher hydronium concentration compared to 0.1 M.

Figure 9b displays a comparison of the average reported diffusion widths in Whatman 40 (this work) and Whatman 5 [47] for the measured green color profile of the reagents (0.1, 0.5 and 1.0 M tartaric acid). The *X*-axis is the concentration based on (g/L) unit, which was converted from the molar (M) unit (concerning the molar mass of tartaric acid) and the *Y*-axis is measured diffusion width based on the millimeter (mm) unit, which was converted from the micrometer (µm) unit.

Moreover, according to Figure 9b, the regression line of Whatman 40 has a higher slope than Whatman 5, therefore, the sensor has improved sensitivity and can capture smaller differences between ionic concentrations of samples. For instance, the Whatman 40 paper displayed a 1012 ± 57.4 µm, 1352 ± 76.3 µm and 1781 ± 65.2 µm of diffusion width for the 0.1 M, 0.5 M and 1.0 M tartaric acid reagents, respectively, while the aforementioned values for the Whatman 5 paper were 975 ± 56.4 µm, 1314 ± 69.1 µm and 1651 ± 34.8 µm, respectively [47]. Furthermore, based on the calculated regression line, the Whatman 40 paper displayed a 5.4 g/L or 35.9 mM limit of detection.

According to Table 2, both papers have a similar basis weight, thickness and density, however, the pore size increased in the case of Whatman 40. Therefore, with increasing pore size the sensitivity of the diffusion sensors increases.

#### 3.2.3. Influence of Papers’ Viscous Resistance on the Turnaround Time of Sensor Results

**Figure 10:**
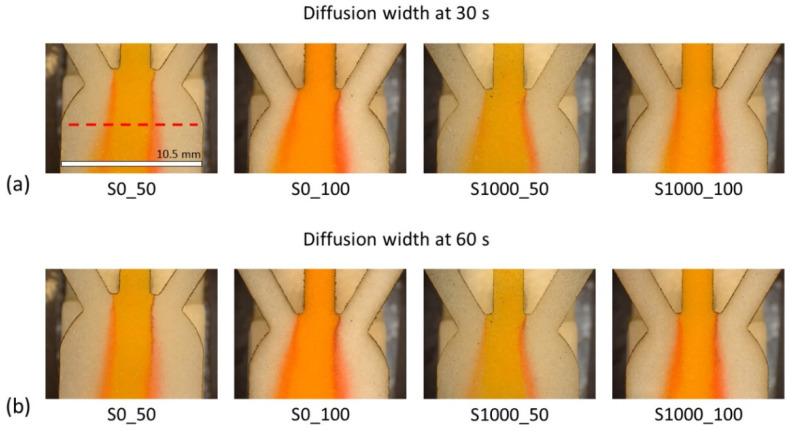
Visual comparison of diffusion development in different lab-made Sisal papers at (**a**) 30 s and (**b**) 60 s after the “Time zero”. The measurement line is displayed as the dashed line. Scale = 10.5 mm.

One of the primary benefits of µPADs is the ability to provide results in a short period. In a diffusion-based sensor, there is a balance between the diffusion of molecules in the transversal direction of the flow and the flow of fluids along the sensor. If there is no flow along the sensor, the diffusion is extremely localized and hard to measure. Heretofore, different lab-made Sisal paper substrates, which had different characteristics (see Table 2), are compared in terms of diffusion width achieved at a given time: 30 s and 60 s after the “Time zero”. Figure 10 summarizes the results when methyl orange (pH indicator) enters through the middle inlet, 0.1 M tartaric acid through the right inlet and wine is flowing through the left inlet.

**Figure 11:**
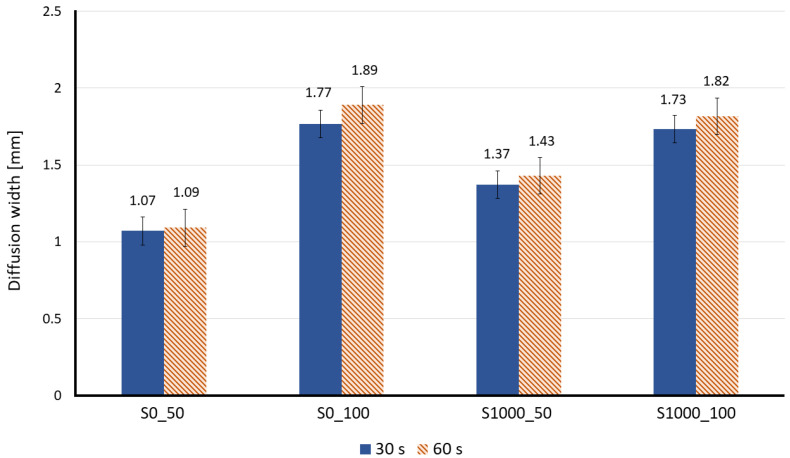
Comparison of measured diffusion width in lab-made Sisal papers at 30 s and 60 s after the “Time zero” for 0.1 M tartaric acid.

While due to high viscous resistance (see Table 5), Whatman grades 5 and 40 at 30 s and 60 s have not generated enough flow to the paper to start measuring, all lab-made Sisal papers with an order of magnitude less in viscous resistance show a measurable response after 30 s and 60 s of the introduction of the reagents, see Figure 11. Sisal papers in 30 s almost achieve a steady state diffusion width and there is not that much difference in diffusion, 30 s later. Therefore, Sisal papers or papers with low viscous resistance are more suitable for point-of-care measurements since they can produce results in a shorter time.

Moreover, the shift of the pH indicator color is quite visible to the naked eye even after 30 s. In lab-made Sisal papers, based on their cellulose fiber characteristics (bigger pore size and length of the cellulose fiber), and higher permeability, the fluid flows faster and decreases the time to generate a co-flow and measuring diffusion. The more intense the diffusion phenomenon is happening, the better mixing with the pH indicator and the sedimentation of methyl orange salts occurs, which establishes a limit on the performance of the sensor. Figure 10b shows that when the viscous resistance is low or the permeability is high (Sisal 0_50 and 0_100) even with the lowest concentration of the solution (0.1 M tartaric acid), the sedimentation is appreciated, and the salts even form fibers, which strand in the pores and as the time passes, they accumulate and prevent further interaction between the reagents.

Between these two papers (Sisal 0_50 and 0_100), even though the permeability is quite similar, the thickness is different (143 µm and 301 µm), respectively. When the thickness is low, the sedimentation increases, and as time passes it can decrease the diffusion velocity of molecules.

**Figure 12:**
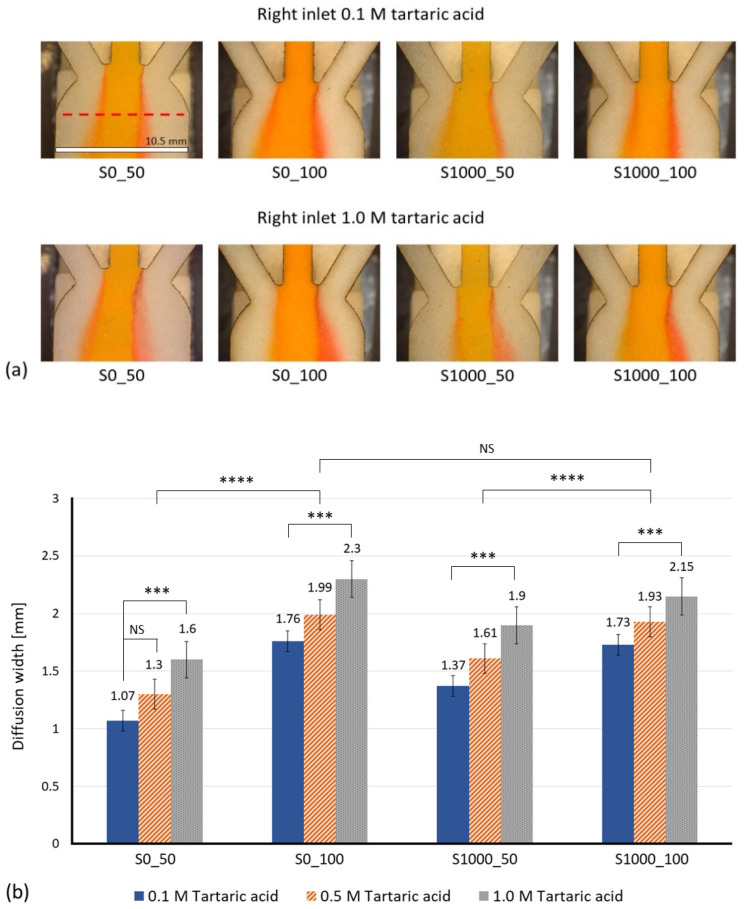
(**a**) Visual comparison of diffusion development in different paper substrates at 30 s after the “Time zero”. The measurement line is shown as the dashed line. Scale Bar is 10.5 mm. (**b**) Measured diffusion width based on the changes in the intensity of the green channel for different concentrations of tartaric acid. The asterisk (*) represents the *p*-value of the statistical test. (*** *p* < 0.001, **** *p* < 0.0001, NS: non-significance).

Figure 12a shows a visual comparison of the diffusion in the different sisal paper substrates for 0.1 M and 1.0 M of tartaric acid at 30 s after the “Time zero”. In all the pictures, wine is entering through the left inlet and methyl orange via the middle inlet. The diffusion width has been experimentally measured.

Figure 12b plots the measured diffusion width for different concentrations of the tartaric acid solution, with a clear linear trend by the increase of species concentration.

**Figure 13:**
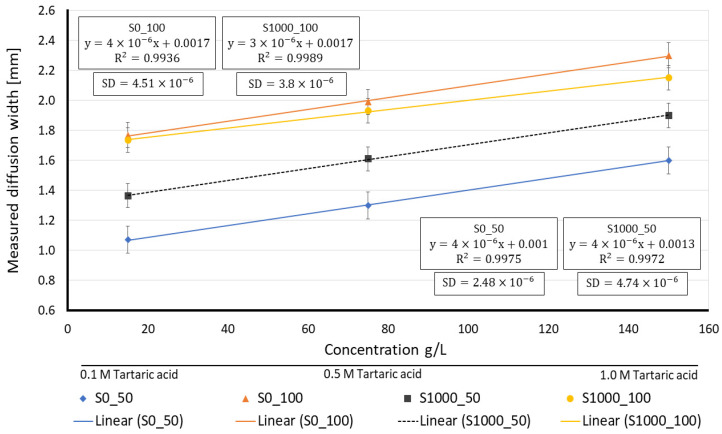
Calibration plot based on the average measured diffusion widths at the measurement line for different concentrations of tartaric acid. (SD = standard deviation).

From the measured diffusion widths over the measurement line at 30 s after the “Time zero”, a calibration plot for each proposed sensor made out of lab-made Sisal papers is obtained, see Figure 13.

According to the results, lab-made Sisal papers with a higher basis weight (100 g/m^2^) show wider diffusion widths compared to the ones with 50 g/m^2^ of basis weight. The reason for the greater difference in diffusion widths between paper substrates with 50 g/m^2^ of basis weight (S0_50 and S1000_50) is due to the sedimentation of the methyl orange salts. The bigger average pore size of S0_50 causes an increase in the fluid velocity through its porous medium and after a certain time, the salts form fibers and prevent further interaction between the reagents. Meanwhile, in S1000_50 (smaller average pore size and higher viscous resistance), the species have more time and space to diffuse. Apart from the pore size and basis weight, another parameter that was not considered in the simulation model, but has an important role in the experimental results, is the thickness. Based on the obtained results, paper substrates with a higher thickness (S0_100 and S1000_100) show wider and more clear diffusion width compared to the papers with less thickness (see Table 2).

The diffusion width of wine Is also measured, being 1024 ± 25.6 µm and 1323 ± 38.7 µm for S0_50 and S1000_50, respectively. As expected, sisal papers with a higher basis weight (100 g/m^2^) showed wider diffusion width for wine and the measured diffusion width of wine for S0_100 was 1726 ± 48.1 µm and S1000_100 showed a diffusion width of 1711 ± 53.5 µm. Moreover, based on the calculated regression line, the S0_50 paper substrate displayed a 6.1 g/L of total acid concentration for the wine sample, whereas the same values for S0_100, S1000_50 and S1000_100 were 6.6, 7.5 and 7.8 g/L, respectively. According to HPLC measurements, the total acid concentration of white wines varies from 5.64 to 10.7 g/L [53], which shows that the results pointed out by this experiment are in agreement with the literature. 

#### 3.2.4. Influence of Basis Weight and Pore Size on the Limit of Detection

In order to see the effect of the papers’ characteristics on the efficiency of the sensor, the limit of detection in all the proposed paper substrates is calculated, as shown in Table 6. For Whatman 40 filter paper (due to the required time to develop a measurable diffusion width), the limit of detection is calculated at 120 s after the “Time zero” and compared with the result presented by Mehrdel et al. [47] for Whatman 5, and for the lab-made Sisal paper substrates, the limit of detection was analyzed at 30 s after the “Time zero”.

**Figure 14:**
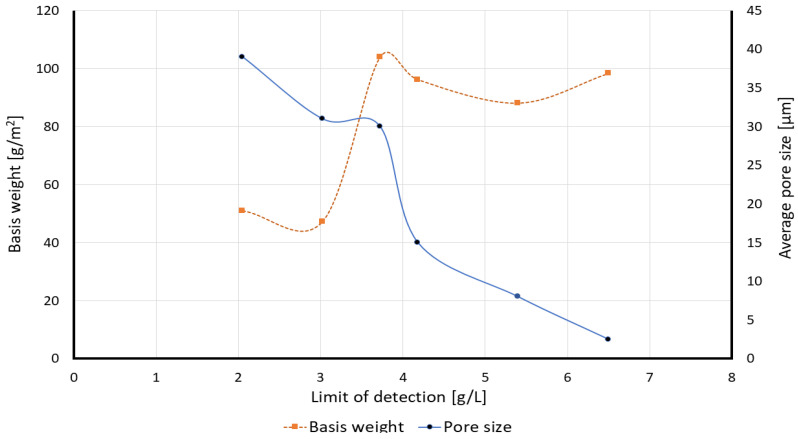
Comparison between the effect of the basis weight and the average pore size on the experimentally obtained limit of detection for different paper substrates.

Based on the calculated limit of detection, it can be concluded that the characterization of the paper substrate can have an important impact on the readout of the results. Paper with a bigger pore size increases the fluid velocity through their porous media (please see Table 5) and therefore less time will be needed to do the analysis. In the case of similar pore sizes, the paper substrate with a lower basis weight can have better accuracy in the results. For instance, S0_50 paper substrate showed a 2.05 g/L or 13.6 mM limit of detection only in 30 s after the “Time zero”. Figure 14 plots the calibration curves based on the effect of the pore size and basis weight on the limit of detection.

The effect of different parameters of papers for performing quantitative measurements which are of great importance for food-quality testing, immunoassays, disease screening in resource-limited areas and point-of-care (POC) diagnostics that enable researchers to perform statistical tests, analyze differences between groups, and determine the effectiveness of treatments, is studied. Prior works have documented the influence of different characteristics of papers by taking advantage of lab-made papers (such as hardwood and softwood fibers) compared to commercial papers. Results pointed out that papers with lower basis weights and higher porosities improved the performance of paper-based devices [45,46]. Casals et al. [37] proposed nonwood cellulose fibers (sisal fibers) and studied the effect of different parameters of these papers, particularly the basis weight and refining process to enhance the paper-based microfluidic devices and the best results were found in non-refined papers with bigger pore size. However, the main focus was on red blood cells (RBCs) tarping and not quantitative analysis.

Apart from the commercial papers (Whatman filter papers), which bring some limitations to having control over different parameters, here we used lab-made Sisal papers to investigate the effect of different characterizations of paper on the performance of the diffusion-based sensors. Lab-made paper with nonwood cellulose fiber (Sisal 0_50) has low basis weight and high porosity (i.e., void fraction). These parameters (particularly bigger pore size which has an important effect on permeability) allow the easy diffusion of molecules through the porous media. Sensors made with such paper deliver high-clarity assay results. Furthermore, since less time is required for the cellulose to be extracted from the sisal plant, as in our lab-made papers, this type of sensor can be more sustainable (Supplementary materials).

## 4. Conclusions

In this work, the effect of paper substrate characteristics (pore size, thickness, basis weight and viscous resistance) on the performance of diffusion paper-based sensors by quantitatively measuring an analyte rather than only detecting its presence, is investigated. The use of a CFD model capable of taking into account certain paper properties (fiber length, fiber diameter, porosity) can help in the substrate selection for the co-flow type of sensors. The experimental tests were in agreement with the numerical results and validated that Whatman filter papers (smaller pore size) required a longer time to produce results than lab-made Sisal papers (bigger pore size). However, certain characteristics not considered in the model such as basis weight and paper thickness are also important.

The experimental results revealed that lab-made paper substrates with bigger pore sizes and lower basis weights, particularly S0_50 with an average pore size of 39 ± 3 µm and basis weight of 50.9 ± 1 g/m^2^, and S1000_50 with an average pore size and basis weight of 31 ± 5 µm and 47.2 ± 0.9 g/m^2^, respectively, showed a better limit of detection than lab-made papers with a higher basis weight (S0_100 and S1000_100) and commercial Whatman papers (smaller pore sizes). In the case of Sisal 0_50 and 1000_50, due to their characteristics and flow behavior through their porous media, it was easier to capture an acceptable diffusion width in a shorter time, which facilitated its measurement and accordingly, enhanced the limit of detection. The sensor made of non-refined lab-made Sisal paper with 50 g/m^2^ of basis weight (S0_50) exhibited an LOD of 13.6 mM when evaluating the ionic strength of wines by comparison to different tartaric acid solutions in 30 s, which is approximately 3 times lower than when using Whatman grade 5 (41.3 mM in 120 s).

However, there are other parameters of interest as well, such as paper additives, paper manufacturing and refining processes, which in turn may affect the fiber length which impacts the capillary tortuosity and uniformity of the paper substrate; these can be considered in order to further optimize the quantitative measurement of the results and tune the generated capillary flow. 

## Figures and Tables

**Table 1:** Dimensions of the substrate.

Property	Value
Total length (L)	30 mm
Main channel width ($W_{\mathrm{ch}}$)	10.5 mm
Measurement line	10.5 mm
Inlet length ($L_{i}$)	9.75 mm
Inlet width ($W_{i}$)	2 mm

**Table 2:** Paper substrate and cellulose fiber characteristics.

Property	Whatman 5	Whatman 40	S0_50	S0_100	S1000_50	S1000_100
Basis weight (g/m^2^)	98.4 ± 0.59 [37]	88.18 ± 0.58 [37]	50.9 ± 1 [38]	104 ± 2 [38]	47.2 ± 0.9 [38]	96.2 ± 2.2 [38]
Density of cellulose (g/m^3^)	1.5 [37]	1.5 [37]	1.45 [37]	1.45 [37]	1.45 [37]	1.45 [37]
Density of the paper (g/m^3^)	0.53 [37]	0.45 [37]	0.36 [38]	0.33 [38]	0.43 [38]	0.47 [38]
Diameter of the cellulose fiber (µm)	19.6 [37]	19.6 [37]	16.03 [37]	16.03 [37]	16.03 [37]	16.03 [37]
Average length of the cellulose fiber (µm)	830 [37]	510 [37]	1500 [38]	1500 [38]	1400 [38]	1400 [38]
Thickness (µm)	186 ± 1 [37]	192 ± 2 [37]	143 ± 11 [38]	301 ± 50 [38]	109 ± 5 [38]	203 ± 5 [38]
Average pore size (µm)	2.5 *	8 *	39 ± 3 [38]	30 ± 7 [38]	31 ± 5 [38]	15 ± 0.7 [38]
Refining(rev)	-	-	0	0	1000	1000

* Nominal particle retention size provided by the manufacturer.

**Table 3:** Physical properties of white wine and tartaric acid at RT (25 °C).

Property	Value
White wine density	1080 kg/m^3^
White wine viscosity	0.00148 kg/m.s [49]
Tartaric acid molar mass	150.078 g/mol
Tartaric acid viscosity	0.00121 kg/m.s (Merck’s catalogue)

**Table 4:** Parameters required to model the porous zone in the Fluent Model.

Paper Type	Porosity (ε)	Permeability $\left(m^{2}\right)$	$D_{eff}\;$ (kg/m.s)
Whatman 5	0.6467	$4.55\times 10^{-15}$	$4.52\times 10^{-6}$
Whatman 40	0.7	$3.02\times 10^{-15}$	$4.89\times 10^{-6}$
S0_50	0.7517	$3.16\times 10^{-14}$	$5.25\times 10^{-6}$
S0_100	0.7724	$4.08\times 10^{-14}$	$5.4\times 10^{-6}$
S1000_50	0.7034	$1.58\times 10^{-14}$	$4.91\times 10^{-6}$
S1000_100	0.6758	$1.17\times 10^{-14}$	$4.72\times 10^{-6}$

**Table 5:** Parameters required to model the porous zone in the Fluent Model.

Paper Type	Viscous Resistance (1/m^2^)	Fluid Velocity (m/s)
Whatman 5	$2.19\times 10^{+14}$	$1.39\times 10^{-4}$
Whatman 40	$3.3\times 10^{+14}$	$5.66\times 10^{-4}$
S0_50	$3.16\times 10^{+13}$	$2.51\times 10^{-3}$
S0_100	$2.45\times 10^{+13}$	$2.3\times 10^{-3}$
S1000_50	$6.32\times 10^{+13}$	$1.78\times 10^{-3}$
S1000_100	$8.51\times 10^{+13}$	$1.43\times 10^{-3}$

**Table 6:** Measured limit of detection for different paper substrates.

Paper Type	Average Pore Size [µm]	Basis Weight [g/m^2^]	LOD at 30 s [g/L]	LOD at 120 s [g/L]
S0_50	39 ± 3	50.9 ± 1	2.05	-
S1000_50	31 ± 5	47.2 ± 0.9	3.02	-
S0_100	30 ± 7	104 ± 2	3.72	-
S1000_100	15 ± 0.7	96.2 ± 2.2	4.18	-
Whatman 40	8	88.18 ± 0.58	-	5.4
Whatman 5	2.5	98.4 ± 0.58	-	6.2 [47]

## Data Availability

Not applicable.

## Supplementary Materials

The following supporting information can be downloaded at: https://www.mdpi.com/article/10.3390/membranes12111074/s1.
